# Mucosal Administration of *Lactobacillus casei* Surface-Displayed HA1 Induces Protective Immune Responses against Avian Influenza A Virus in Mice

**DOI:** 10.4014/jmb.2307.07040

**Published:** 2023-10-31

**Authors:** Dung T. Huynh, W.A. Gayan Chathuranga, Kiramage Chathuranga, Jong-Soo Lee, Chul-Joong Kim

**Affiliations:** College of Veterinary Medicine, Chungnam National University, Daejeon 34314, Republic of Korea

**Keywords:** Avian influenza, HA1, *Lactobacillus casei*, poly γ-glutamic acid synthetase A, surface display, mucosal delivery

## Abstract

Avian influenza is a serious threat to both public health and the poultry industry worldwide. This respiratory virus can be combated by eliciting robust immune responses at the site of infection through mucosal immunization. Recombinant probiotics, specifically lactic acid bacteria, are safe and effective carriers for mucosal vaccines. In this study, we engineered recombinant fusion protein by fusing the hemagglutinin 1 (HA1) subunit of the A/Aquatic bird/Korea/W81/2005 (H5N2) with the *Bacillus subtilis* poly γ-glutamic acid synthetase A (pgsA) at the surface of *Lactobacillus casei* (pgsA-HA1/*L. casei*). Using subcellular fractionation and flow cytometry we confirmed the surface localization of this fusion protein. Mucosal administration of pgsA-HA1/*L. casei* in mice resulted in significant levels of HA1-specific serum IgG, mucosal IgA and neutralizing antibodies against the H5N2 virus. Additionally, pgsA-HA1/*L. casei*-induced systemic and local cell-mediated immune responses specific to HA1, as evidenced by an increased number of IFN-γ and IL-4 secreting cells in the spleens and higher levels of IL-4 in the local lymphocyte supernatants. Finally, mice inoculated with pgsA-HA1/*L. casei* were protected against a 10LD_50_ dose of the homologous mouse-adapted H5N2 virus. These results suggest that mucosal immunization with *L. casei* displaying HA1 on its surface could be a potential strategy for developing a mucosal vaccine against other H5 subtype viruses.

## Introduction

Avian influenza (AI), caused by AI viruses (AIVs) from *Orthomyxoviridae* family [1], is a highly contagious respiratory disease in poultry [2]. Based on their pathogenicity, AIVs are classified into two groups highly pathogenic avian influenza viruses (HPAIVs) and low pathogenic avian influenza viruses (LPAIVs). HPAIVs pose a greater threat to poultry [3]. LPAIVs can mutate into HPAIVs, increasing the risk of outbreaks [4-6]. Additionally, there is an increase in the number of direct transmission cases of AIVs from birds to humans, which could lead to sudden epidemics [7, 8].

HPAIVs of the H5 subtype, derived from the A/Goose/Guangdong/1/1996 (H5N1) lineage, known as H5Nx viruses, have become a growing concern due to their increasing prevalence and their potential to cause human infections [9]. These viruses have evolved into ten genetic clades [10]. Among the ten clades, clade-2 viruses are successively dominant and endemic in many countries [11]. Outbreaks of H5N5 and H5N2 HPAIVs have been reported in Asia, Europe, and North America since 2008 [12-15]. In addition, H5N8 viruses from clade 2.3.4.4 were primarily reported in Korea in early 2014 and spread globally in many countries [16]. In 2014, a novel H5N6 reassortant-caused human infection was first reported in China [17]. The recurrence of H5N2 LPAIV raises concerns about the exchange of genetic characteristics between divergent isolates [18-20]. Although H5Nx viruses are currently unable to efficiently transmit among humans [21, 22], their continued evolution and lack of population-level immunity make them a potential pandemic threat [23]. Therefore, the development of an effective H5 subtype AI vaccine is urgently needed.

The respiratory tract mucosa serves as the site of entry and replication of the AI virus as well as the front line of defense against infections [24]. However, current parenteral AI vaccine modalities generally fail to induce local immune responses [25, 26], which is why mucosally administered vaccines are more effective [27]. These mucosal vaccines can elicit both local and systemic immunity but, local barriers remain a bottleneck of antigen uptake by microfold cells and professional antigen-presenting cells [28]. To overcome this issue, it is necessary to investigate safer and more efficient mucosal vaccine carriers for clinical use [29]. *Lactobacillus* species is an attractive delivery platform for mucosal vaccines due to their inherent safety and intrinsic adjuvant properties. Many studies have shown that mucosally administered *Lactobacillus* triggered local IgA and systemic immune responses, including T and NK cell activity by activating internalization and maturation of antigen-presenting cells in draining lymph nodes and spleen [30-32].

In previous studies, we successfully engineered the *Bacillus subtilis* transmembrane protein poly-γ-glutamate synthetase A (pgsA) to present multiple heterologous antigens at the surface of *L. casei* [33-36]. We also demonstrated that *L. casei* surface-displayed universal vaccine candidate sM2HA2, a fusion protein of conserved matrix protein 2 (sM2) and the stalk domain of hemagglutinin 2 (HA2), induced strong sM2HA2-specific humoral and cellular immune responses following mucosal immunization [35, 36]. However, we observed that this approach did not provide complete protection in mice infected with H5 subtype viruses. To address this issue, we targeted the immunodominant hemagglutinin 1 (HA1) subunit that constitutes the membrane-distal globular domain of HA, which contains the sialic acid receptor-binding site and most of the determinants recognized by virus-neutralizing antibodies [37]. Immunization with recombinant H5N1-derived HA1 provided effective protection against heterologous influenza virus strains [38, 39]. Thus, incorporating HA1 as a vaccine target in our mucosal vaccine platform could potentially enhance protection against H5 subtype influenza viruses.

In this study, we selected the HA1 domain (residues 17 to 330) of A/aquatic bird/Korea/W81/2005(H5N2) as a representative immunogen for AI H5 subtypes and investigated the immunogenicity of HA1 which was displayed on the surface of *L. casei* (pgsA-HA1/*L. casei*) in the BALB/c mouse model. The results showed that oral and intranasal immunization of mice with pgsA-HA1/*L. casei* elicited protective immune responses against the homologous mouse-adapted H5N2 virus. The results suggest that recombinant *L. casei* can serve as a promising delivery platform for the development of mucosal vaccines against AI viruses and other pathogens.

## Materials and Methods

### pgsA-HA1/*L. casei* Construction and Expression

The plasmid encoding the H5N2/HA sequence was kindly provided by Dr. Young-Ki Choi (Chungbuk National University, South Korea). The plasmid pKV-Pald-PgsA, harboring the pgsA genes of *Bacillus subtilis*, was used to construct the surface display plasmid, which was a kind gift from the Bioleaders Corporation (South Korea). HA1 fragment spanning residues 17 to 330 was amplified by PCR using 5’-GGATCCGACCAAATTTGCATTGG-3’-forward primer and 5’-GTCGACTTACCCAAATAGTCCTCTTG-3’-reverse primer with the H5N2/HA plasmid as a template. The insert was then digested by BamHI/SalI restriction enzymes (Takara, South Korea) and cloned into the linear expression vector pKV-Pald-pgsA to yield pKV-Pald-pgsA-HA1. The plasmid was subsequently verified by DNA sequencing (Bionics, South Korea).

The plasmids pKV-Pald-pgsA (empty plasmid) and pKV-Pald-pgsA-HA1 were introduced into *Lactobacillus casei* L525 cells by electroporation for expression (so-called pgsA/*L. casei* and pgsA-HA1/*L. casei*, respectively) following a previously described protocol [40], In brief, 1 ml pre-culture of *L. casei* L525 was inoculated into 50 ml of MRS media (BD Biosciences, USA) and grown without aeration at 30°C until the culture reached an optical density at 600 nm up to 2-2.5. Subsequently, the growth was halted by cooling the culture on ice for 15 min, and the cells were harvested by centrifugation (6000 × g, 2 min, 4°C). The cell pellet was washed three times with ice-cold ultra-pure water, the final cell pellet was suspended in 1 ml of ice-cold ultra-pure water. Next plasmid DNA (250 ng/transformation) was combined with 100 μl cell suspension, transferred to a sterile prechilled electroporation cuvette (with a 2 mm gap), and subjected to electroporation under the following conditions: voltage of 2000V, parallel resistance of 400 Ω, capacitance of 25 μF, field strength of 10 kV/cm), and time constant of 5 ms using the Gene Pulser Xcell electroporation system (BioRad, USA). In accordance with a previous protocol [35], pgsA/*L. casei* and pgsA-HA1/*L. casei* cells were fractionated after being cultured for 48 h at 30°C in MRS broth with erythromycin (16 μg/ml) (Merck, Germany). Subsequently, immunoblotting analysis was performed using rabbit anti-pgsA (1:3000) and mouse anti-H5N2 (1:1000) polyclonal antibodies (home-grown) and horseradish peroxidase (HRP)-conjugated rabbit or mouse anti-IgG (1:3000) (Cell Signaling Technology, USA). The WEST-ZOL plus immunoblotting blotting detection system (iNtRON Biotechnology, South Korea) was used for visualization of immunoblotting with enhanced chemiluminescence (ECL) detection system (ECL-GE Healthcare, United Kingdom).

To perform fluorescence-activated cell sorting (FACS), recombinant *L. casei* containing pgsA, pgsA-HA1 were harvested and incubated overnight with mouse anti-H5N2 (1:1000). Subsequently, the cells were incubated with Cy3-conjugated donkey mouse anti-IgG antibody (Jackson immunoresearch Inc, Pennsylvania, USA) for 2 h at room temperature (RT). Finally, a total of 1 × 10^5^ cells were analyzed by flow cytometry (Becton Dickinson, USA).

### Mouse Experimental Schedule, Sample Collection, and Virus Challenge

Specific pathogen-free female BALB/c mice (6-week age) were purchased from Samtako (South Korea) and maintained in a ventilated milieu with ad libitum access to water and food. The room was maintained at a temperature of 18-23°C, relative humidity of 50-60%, and a 12 h light/dark cycle. All mice were allowed to acclimate for 7 days before the start of the experiment. All experiments were conducted under appropriate conditions with the approval of the Institutional Animal Care and Use Committee of Chungnam National University (approval number CNU-00432). In all intranasal immunization and challenge experiments, mice were anesthetized with intraperitoneal administration of avertin (2.5%) at a dosage of 0.015 ml/g bodyweight.

The study consisted of two sets of mice, one for oral and one for intranasal immunization. Each set was divided into three experimental groups, consisting of 19 mice (eight for characterization of humoral and cellular immune responses, five for survival analysis and six for lung virus titers at 3 and 5 days post-challenge (dpc). The mice were immunized with pgsA-HA1/*L. casei* at a dose of 10^10^ colony forming unit (CFU) in 100 μl of PBS via oral gavage on days 0-2, 7-9 and 21-23 or at a dose of 10^9^ CFU in 20 μl of PBS via pipette drop to the nostrils of anesthetized mice on days 0-2, 7-9 and 21. The control mice in the oral and intranasal groups were administered the same dosage of *L. casei* harboring pgsA/*L. casei* or same volume of PBS. Blood and feces samples were collected on days -1 and 14, and 28, and the lungs and small intestines were collected on day 28. The sera were separated from whole blood by centrifugation (12,000 ×*g*, 5 min, 4°C). Feces, lungs and intestines were homogenized in PBS containing 1 mM phenylmethylsulfonyl fluoride, and the supernatants were collected by centrifugation (12,000 ×*g*, 15 min, and 4°C).

Spleens, cervical lymph nodes, mesenteric LNs and Peyer’s patches LNs were aseptically collected on day 28 and stored in RPMI media (PAN Biotech, Germany). All tissue samples were separated through a 70 μm cell strainer filter (SPL Life science, South Korea), and lymphocytes from cervical, mesenteric and Peyer’s patches LNs were kept on ice. Splenocytes from the spleens were harvested after the lysis of red blood cells with ammonium-chloride-potassium buffer. The cells were then suspended in complete RPMI media containing 10% fetal bovine serum (FBS) with 1% antimycotic and antibiotic (Gibco, USA). A synthetic peptide containing the conserved epitope of H5-subtype hemagglutinin (CNTKCQTPMGAINSS) [41] was synthesized by Peptron Inc. (South Korea). This synthetic peptide was used for in vitro re-stimulation assays at a concentration of 5 μg/well.

To assess the protective efficacy of the pgsA-HA1/*L. casei* vaccine, mice were challenged with a 10LD_50_ of the mouse-adapted A/Aquatic bird/Korea/W81/2005(H5N2) virus via intranasal inoculation in 20 μl of PBS. Virus titer in the lungs was determined by sacrificing six mice from each group on 3 and 5 dpc. The remaining five mice from each group were monitored daily for weight loss and survival for a total of 12 days. Mice that lost 25% of their body weight were considered to have reached the humane endpoint and were euthanized using CO_2_ inhalation. Efforts were made to minimize suffering and all surviving mice were humanely euthanized after final monitoring.

### ELISA

Antibodies specific to HA1 were determined using an indirect enzyme-linked immunosorbent assay (ELISA). To coat the 96-well immunosorbent plates (Corning, USA), the above synthetic peptide (500 ng/well) was added and allowed to incubate overnight at 4°C. Following, 2 h of blocking at RT with 10% skim. Serum (1:50) or supernatants of homogenized tissues or feces (1:200) were added to the plates and incubated for 2 h at 37°C. The plates were then incubated with secondary HRP-conjugated goat anti-mouse IgG, -IgG1, IgG2a, -IgA antibodies (diluted 1:3000; Sigma, USA) for 2 h at 37°C. The plates were then incubated in the dark for 10 min with a mixed substrate solution of 3,3’,5,5’-Tetramethylbenzidine (TMB) and H_2_O_2_. Finally, the reaction was stopped by adding 2N-H_2_SO_4_, and optical density values at 450 nm wavelength were measured with an Apollo ELISA Reader (Berthold technologies, Germany).

Sandwich ELISA was used to evaluate the levels of antigen-specific interleukin-4 (IL-4) secretion in the local LNs. For the in vitro stimulation assay, 1 × 10^6^ lymphocytes from cervical, mesenteric and Peyer’s patches LNs were incubated with the above synthetic peptide in complete RPMI media at 37°C with 5% CO_2_ for 72 h. The culture supernatants were collected, centrifuged and stored at -20°C for further analysis. The production of IL-4 was measured using a cytokine ELISA kit (BD Biosciences, USA) following the manufacturer's instructions.

### ELISPOT

The HA1specific cellular immune responses were evaluated by using an ELISPOT assay with the mouse IFN-γ ELISPOT set and mouse IL-4 ELISPOT set according to the manufacturers’ specifications (BD Bioscience, USA). First, 96-well ELISPOT plates were coated with anti-mouse IFN-γ or anti-IL-4 capture antibodies (5 μg/ml) in PBS and incubated at 4°C overnight. Then plates were blocked for 2 h at RT with 200 μl/well complete RPMI media. Next, 1 × 10^6^ splenocytes were added to each well and incubated for 48 h at 37°C with 5% CO_2_ in complete RPMI media containing the above synthetic peptide (5 μg/well), complete RPMI media (negative control) or RPMI media with 5 μg/ml phytohaemagglutinin (positive control) (Invitrogen, USA). The cells were then discarded from the plates and treated with biotinylated anti-mouse IFN-γ and IL-4 antibodies, streptavidin HRP and AEC substrate solution. The substrate reaction was terminated by washing with deionized water. Finally, the spots were enumerated using the CTL-Immunospot S5 UV analyzer (Cellular Technologies, USA).

### Lung Virus Titer

The lungs were aseptically collected to determine virus titers using 50% tissue culture infectious dose (TCID_50_) as previously described [35]. In brief, lung tissues were homogenized in sterilized PBS containing 1% antibiotic and antimycotic solution, followed by centrifugation (12,000 ×*g*, 15 min, and 4°C) to remove tissue debris. Ten-fold serial dilution of the samples was added to confluent MDCK cells and incubated at 37°C with 5% CO_2_ for 1h. The media was replaced with L-1-tosylamide-2-phenylethyl chloromethyl ketone (TPCK) trypsin (Thermo Fisher Scientific, USA) containing serum-free media, the titers of each serially diluted sample were examined using a hemagglutination assay (HA) test. The lung virus titers were expressed as a TCID_50_ using the Reed-Muench method [42].

### Serum Neutralization Test

The level of H5N2-specific neutralizing antibodies in sera was determined by conducting a serum neutralization test with modification to the protocol described previously [43]. In 96-well microliter plates, 50 μl of 2-fold serial dilution of receptor-destroying enzyme (Denka Seiken, Japan) was treated and then inactivated serum at 56°C for 30 min in FBS free DMEM was mixed with 50 μl of 100 TCID_50_ of Vero cell adapted H5N2 virus. This mixture was then incubated at 37°C for 1h, 5 × 10^3^/100 μl of Vero cells were added to each well, and the plates were incubated at 37°C with 5% CO_2_ for 4 days. The virus-induced cytopathic effect was examined and the reciprocal of the highest serum dilution at which cytopathic effect could be observed was used to determine the neutralizing antibody titers.

### Statistical Analysis

The results are reported as the mean values with standard deviations (S.D.). Discrepancies between groups were analyzed using analysis of variance (ANOVA) followed by Tukey's multiple comparison test. Survival rates were compared using the log-rank test, using GraphPad Prism 6 software. The threshold for statistically significance was set at *p* < 0.05.

## Results

### Construction, Expression and Surface Localization of pgsA-HA1

**Fig. 1:**
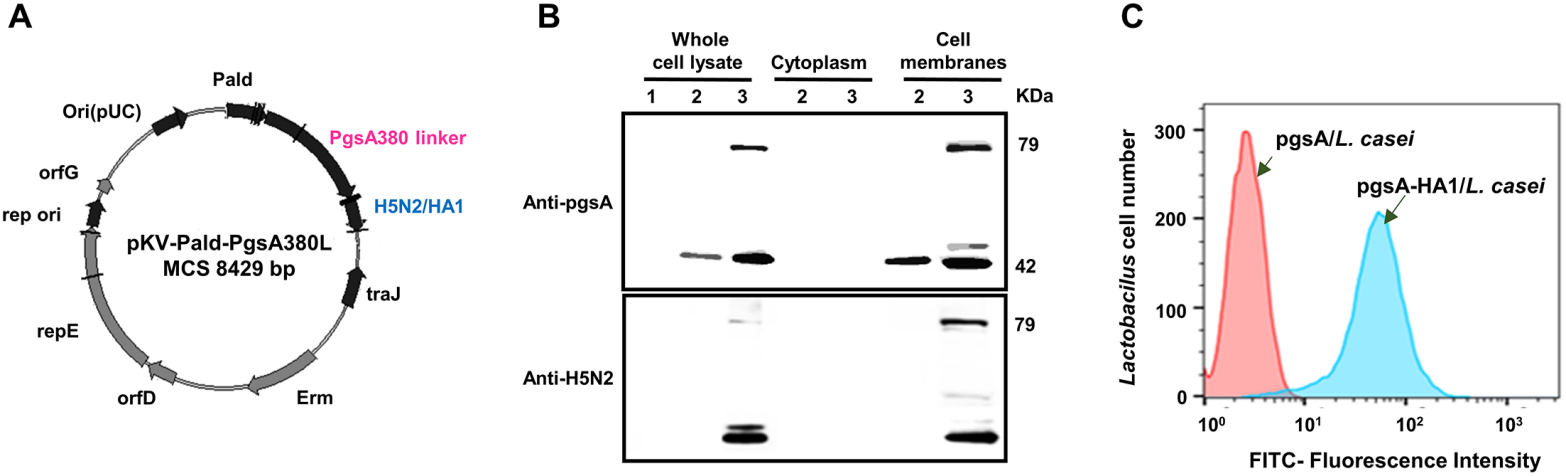
Antigen construction and expression. (**A**) Schematic depiction of the pKV-Pald-pgsA-HA1 plasmid. (**B**) The immunoblotting of the fractionated recombinant *L. casei*, pgsA/*L. casei* and pgsA-HA1/*L. casei* using anti-pgsA and anti-H5N2 polyclonal antibodies. Lanes 1, 2, and 3 illustrated *L. casei*, pgsA/*L. casei* and pgsA-HA1/*L. casei*, respectively. (**C**) FACS analysis. The recombinant pgsA/*L. casei* and pgsA-HA1/*L. casei* cells were probed with mouse anti-H5N2 antibody, followed by Cy3-conjugated donkey mouse anti-IgG antibody.

Initially, we constructed a pKV-Pald-pgsA-HA1 plasmid containing a fusion gene of pgsA-HA1 (Fig. 1A). *L. casei* cells were then electroporated for expression of pgsA or pgsA-HA1 recombinant antigens. The subcellular fractionation was conducted to verify the proper localization of recombinant HA1 on the membranes of *L. casei*. Differential centrifugation was used to separate cytoplasmic and cell membrane fractions and fractions were analyzed by immunoblotting using anti-pgsA and anti-H5N2 antibodies. Virtually no pgsA or pgsA-HA1 was detected in the negative control sample (Fig. 1B, lane 1). As expected, pgsA-HA1 fusion protein was detected at the expected molecular weight of approximately 79 kDa. Both recombinant pgsA and pgsA-HA1 were confirmed in whole-cell lysates and cell membrane fractions but not in the cytoplasmic fractions (Fig. 1B lanes 2 and 3). However, some lower molecular weight bands were observed in the whole-cell lysates and cell membrane fractions of the pgsA-HA1/*L. casei*, which may have resulted from degradation.

Upon proper localization to the *L. casei* membranes, pgsA-HA1 fusion protein was expected to display at the cell surface. To confirm the surface accessibility of recombinant pgsA-HA1, *L. casei* cells were subjected to FACS analysis. The pgsA-HA1/*L. casei* reacted with mouse anti-H5N2 antiserum, followed by Cy3-conjugated mouse anti-IgG antibody (Jackson ImmunoResearch, USA). FACS analysis results demonstrated the successful surface exposure of pgsA-HA1 fusion protein (Fig. 1C). These findings confirmed that recombinant HA1 was successfully expressed and displayed at the cell surface of *L. casei* using pgsA as a membrane anchor protein.

### Recombinant pgsA-HA1/*L. casei* Induced systemic and local humoral immune responses

**Fig. 2:**
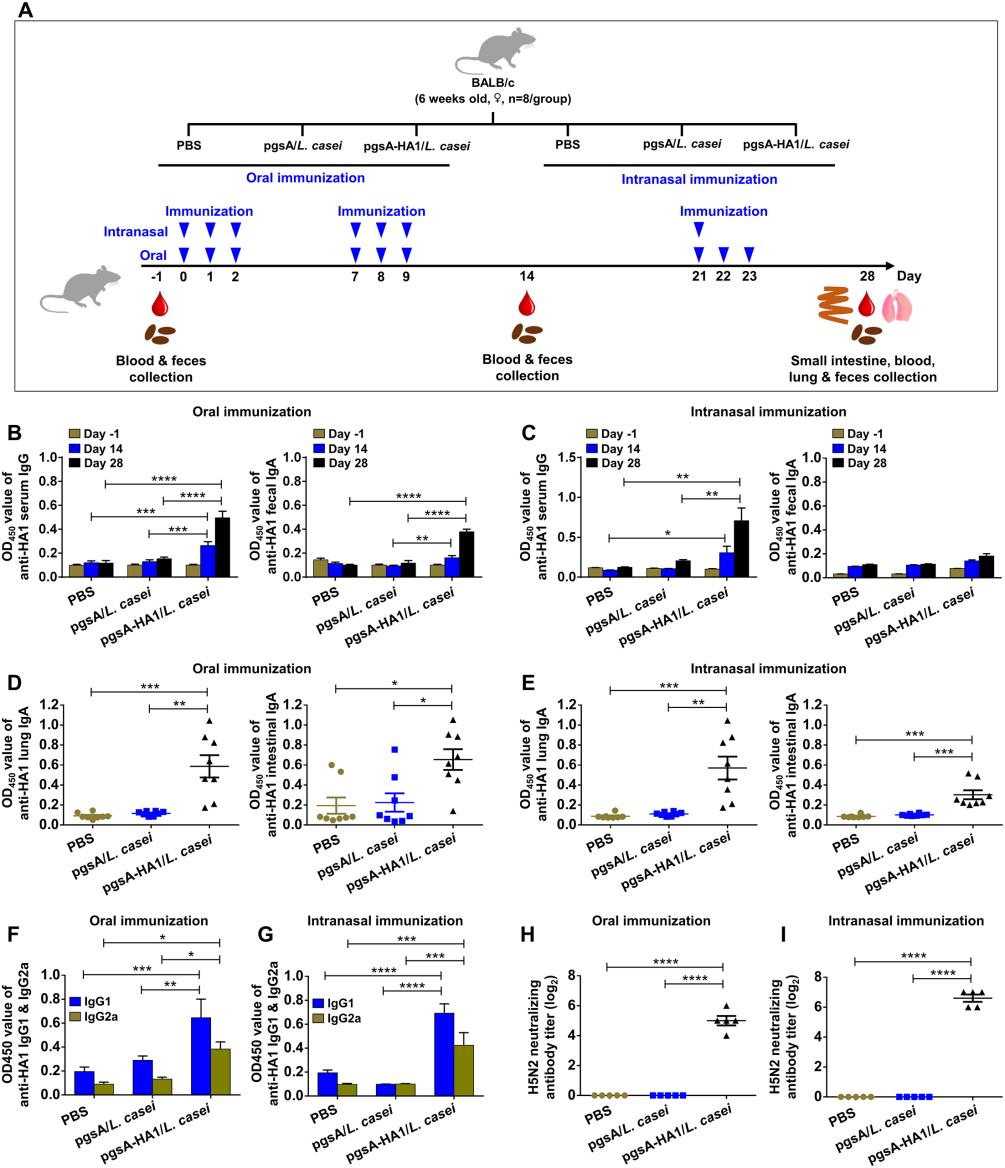
Evaluation of the antigen-specific humoral immune responses induced by pgsA-HA1/*L. casei*. Mice were grouped as mentioned in materials and methods, then immunized at days 0 to 2, 7 to 9, and 21 to 23 orally and intranasally. Blood and feces samples were collected on days -1, 14, and 28. Lung and small intestine samples were collected on day 28 after immunization. Induction of HA1-specific humoral immune responses by mucosal immunization of pgsA-HA1/*L. casei* were determined by indirect ELISA and virus neutralization assay. (**A**) Schematic depiction of mouse experiment strategy. (**B**) HA1- specific serum IgG titers (left panel) and fecal IgA titers (right panel) in the orally immunized groups. (**C**) Similarly, in the intranasally immunized groups, HA1-specific serum IgG titers (left panel) and fecal IgA titers (right panel). (**D**) HA1-specific IgA titers in the lungs (left panel) and the small intestines (right panel) in the orally immunized groups. (**E**) Similarly, HA1- specific IgA titers in the lungs (left panel) and the small intestines (right panel) in the intranasally immunized groups. (**F**) HA1- specific IgG1 and IgG2a titers in the orally immunized groups. (**G**) Reciprocals of virus-neutralizing antibody titers specific to the H5N2 virus in the orally immunized groups. (**H**) HA1-specific IgG1 and IgG2a titers in the intranasally immunized groups. (**I**) Reciprocals of virus-neutralizing antibody titers specific to the H5N2 virus in the intranasally immunized groups. The bars denote the means ± SD. Statistical analyses were performed using two-way ANOVA with Tukey's multiple comparisons test., *p < 0.05, **p < 0.01, ***p < 0.001, ****p < 0.0001.

To assess the humoral immune responses of pgsA-HA1/*L. casei*, mice were immunized through oral and intranasal routes as per a specific scheme (Fig. 2A). Two control groups (PBS and pgsA/*L. casei*) were used. Serum and fecal samples were collected on -1, 14, and 28 days and analyzed via ELISA. Serum collected on 14 days showed a relatively low absorbance level for IgG specific to HA1. However, on day 28, high levels of absorbance for IgG specific to HA1 were observed in the pgsA-HA1/*L. casei* immunized mice, in both orally (Fig. 2B, left panel) and intranasally (Fig. 2C, left panel) inoculated groups. Oral immunization with pgsA-HA1/*L. casei* induced significant levels of fecal IgA levels specific to HA1 (Fig. 2B, right panel), while no significant difference in fecal IgA antibodies was observed in all groups with intranasal immunization (Fig. 2C, right panel). IgA secreted from the mucosa is an essential indicator of the local immune response to natural infection and studies have shown that secretory IgA responses in mice were shown to be involved in heterosubtypic cross-protection [44]. Recombinant *L. casei* surface-expressing pgsA-HA1 evoked significantly increased levels of HA1-specific local IgA titers in the lungs and small intestines compared to the controls both orally (Fig. 2D) and intranasally (Fig. 2E) immunized groups. These data demonstrate that *L. casei* surface-displayed HA1 antigen effectively stimulated systemic IgG and local IgA antibodies.

To further analyze the antigen-specific systemic humoral immune responses induced by pgsA-HA1/*L. casei* after mucosal immunization, the pattern of IgG-isotypes was examined using ELISA. Oral (Fig. 2F) and intranasal (Fig. 2G), administration of recombinant pgsA-HA1/*L. casei* induced significantly high levels of HA1-specific IgG1 and IgG2a compared to the control groups. These results indicated that pgsA-HA1/*L. casei* induced mixed Th1/Th2-CD4^+^ immune responses, which promotes IgG class-switching and secretion of IgA as observed earlier.

### pgsA-HA1/*L. casei* Enhanced H5N2 Specific Virus-Neutralizing Antibodies

Virus-neutralizing activity is a direct and sensitive measure for functional antibodies [45]. To examine whether the pgsA-HA1/*L. casei* can induce anti-H5N2 specific neutralizing antibodies, serum neutralization assay was performed in the Vero cells. The neutralizing antibodies were determined as the highest serum dilution that inhibited the cytopathic effect in Vero cells. Both oral and intranasal immunization with pgsA-HA1/*L. casei* induced significantly higher titers of H5N2 neutralizing antibodies than those inoculated with pgsA/*L. casei* or PBS groups (Fig. 2H and 2I). Notably intranasal immunization using pgsA-HA1/*L. casei* appeared relatively higher titers of neutralizing antibodies compared to oral immunization (Fig. 2H and 2I), indicating the benefits of intranasal administration in inducing systemic humoral responses.

### pgsA-HA1/*L. casei* Induces Potential HA1-Specific Cellular Immune Response

**Fig. 3:**
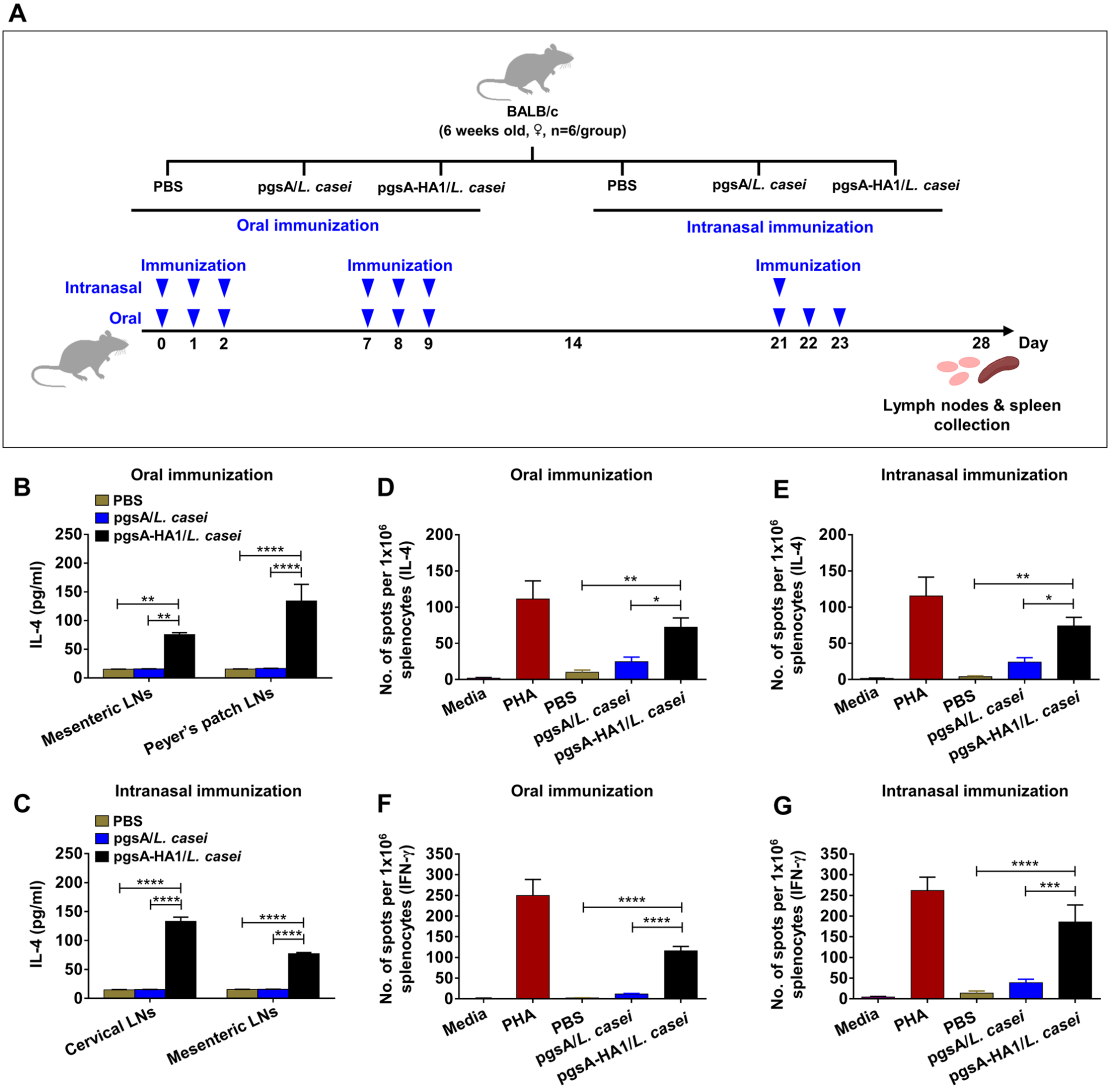
Evaluation of potential antigen-specific cellular immune responses induced by pgsA-HA1/*L. casei*. Mice were grouped as mentioned in materials and methods, then immunized at days 0 to 2, 7 to 9, and 21 to 23 orally and intranasally. Local LNs and spleen lymphocytes were collected on day 28 after immunization. Induction of potential HA1- specific cellular immune responses by mucosal immunization of pgsA-HA1/*L. casei* was determined by cytokine ELISA and ELISPOT assays. (**A**) Schematic depiction of mouse experiment strategy. (**B**) HA1-protein specific interleukin-4 (IL-4) production by mesenteric and Peyer’s patches LN lymphocytes (1 × 10^6^ cells) in the orally immunized groups by ELISA. (**C**) HA1-specific interleukin-4 (IL-4) production by cervical and mesenteric LN lymphocytes (1 × 10^6^ cells) in the intranasally immunized groups by ELISA. (**D**) and (**E**) HA1-specific IL-4 spots forming splenic lymphocytes (1 × 10^6^ cells) in the orally and intranasally immunized groups by ELISPOT, respectively. (**F**) and (**G**) HA1- specific interferon-γ (IFN-γ) spots forming splenic lymphocytes (1 × 10^6^ cells) in the orally and intranasally immunized groups by ELISPOT, respectively. PHA: Phytohaemagglutinin. The bars denote the means ± SD. Statistical analyses were performed using two-way ANOVA with Tukey's multiple comparisons test., *p < 0.05, **p < 0.01, ***p < 0.001, ****p < 0.0001.

In addition to humoral immune responses, cellular immune responses are also important for influenza clearing [46]. In this study, mice were immunized through oral and intranasal routes per the specific scheme (Fig. 3A). To evaluate potential antigen-specific T cell responses, in vitro lymphocyte restimulation assays were conducted using cells from cervical, mesenteric and Peyer’s patches LNs on day 28 after immunization. The mesenteric and Peyer’s patches LN cells were isolated from the mice orally inoculated with pgsA-HA1/*L. casei* showed significantly higher IL-4 production than the control groups (Fig. 3B). In mice intranasally immunized with pgsA-HA1/*L. casei* dramatic increases in local IL-4 levels were observed in cervical and mesenteric LN cells (Fig. 3C).

**Fig. 4:**
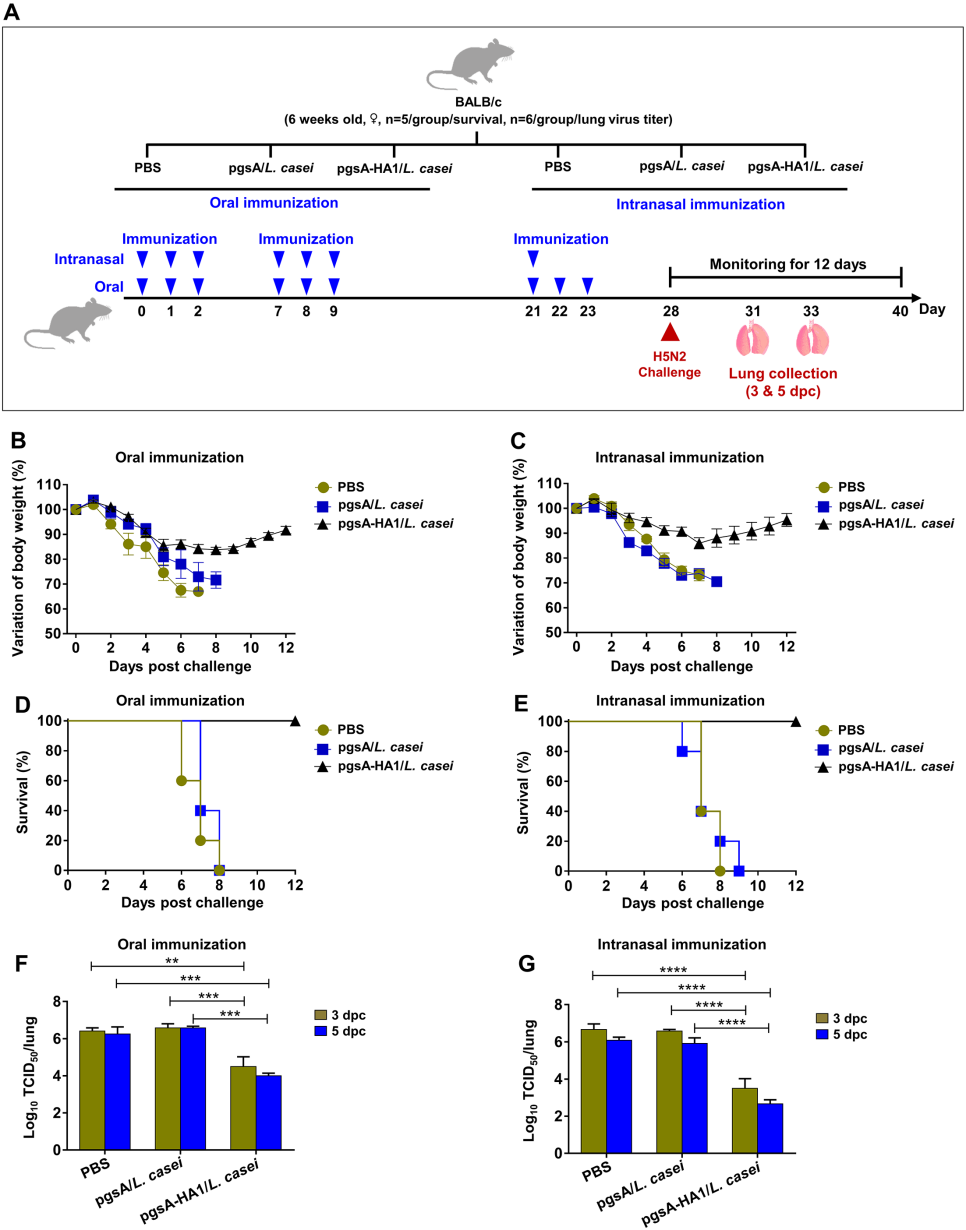
Protective efficacy of the pgsA-HA1/*L. casei* against lethal H5N2 infection. Mice were grouped as mentioned in materials and methods, then immunized at days 0 to 2, 7 to 9, and 21 to 23 orally and intranasally. Mice were intranasally challenged with 10LD_50_ of mouse-adapted A/Aquatic bird/Korea/W81/2005 (H5N2). After the challenge, changes in body weight and proportion of survival were monitored for 12 days. Lungs were aseptically collected on days 3 and 5 postchallenge; virus titers in the lung tissues were investigated by TCID_50_ in MDCK cells following the infection with H5N2. (**A**) Schematic depiction of mouse experiment strategy. (**B**) and (**C**) Changes in body weight and (**D**) and (**E**) survival rates of orally and intranasally immunized groups, respectively. (**F**) and (**G**) Virus titers in the lung tissues in orally and intranasally immunized groups, respectively. The bars denote the means ± SD. Statistical analyses were performed using two-way ANOVA with Tukey's multiple comparisons test., **p < 0.01, ***p < 0.001, ****p < 0.0001.

Furthermore, cellular immune responses to pgsA-HA1/*L. casei* were confirmed by measuring both IL-4 and IFN-γ secretion levels from splenocytes using the ELISPOT assay on day 28 after immunization. Splenic lymphocytes isolated from mice immunized with pgsA-HA1/*L. casei* through both oral and intranasal routes showed significantly higher levels of systemic IL-4 (Fig. 4D and 4E) and IFN-γ (Fig. 4F and 4G) production than mice administered pgsA/*L. casei* or PBS. Taken together, these data suggest that pgsA-HA1/*L. casei* effectively induced local and systemic Th1/Th2 cytokine profiles and also confirmed mixed Th1/Th2-dependent cellular and humoral immune responses as observed above (Fig. 2F and 2G).

### Mucosal Immunization of pgsA-HA1/*L. casei* Showed Protection against Lethal H5N2 Virus Challenge

Given that pgsA-HA1/*L. casei* was able to induce significant humoral and potential cellular immune responses, we sought to determine whether mucosal immunization with this vaccine would confer protection against lethal homologous H5N2 virus infection. To test the efficacy of the mucosal vaccine, we challenged immunized mice intranasally with a 10LD_50_ dose of mouse-adapted A/Aquatic bird/Korea/W81/2005(H5N2) and monitored changes in body weight and percentage survival for 12 days (Fig. 4A). The mice were orally immunized with pgsA-HA1/*L. casei* showed a 15% reduction in body weight, whereas the intranasally immunized mice showed a 7%reduction at 7 dpc. Both intranasally and orally immunized mice initiated recovery at 7 and 9 dpc, respectively, and completely recovered at 12 dpc (Fig. 4B and 4C). Both oral and intranasal inoculations with the pgsA-HA1/*L. casei* triggered 100% protection against the lethal challenge of the H5N2 virus, whereas all control group mice lost a significant magnitude of body weight and died by 8 to 9 dpc (Fig. 4D and 4E). However, the findings also indicated that intranasal immunization of pgsA-HA1/*L. casei* evoked more potent protection against the H5N2 virus infection.

### Immunization with pgsA-HA1/*L. casei* Reduced Lung Virus Titers after H5N2 Challenge

Viral load in the lungs after infection is a reliable indicator of vaccine protection efficacy. The viral load in the lungs was quantified using the TCID_50_ method. pgsA-*L. casei* and PBS vaccine group had higher lung virus titers compared to the pgsA-HA1/*L. casei* vaccinated group. Notably, the pgsA-HA1/*L. casei* orally inoculated group showed a significant decrease in lung virus titers in comparison to the control group (Fig. 4F). However, this reduction was lower than that observed in the respective intranasally immunized group (Fig. 4G). These data demonstrate that pgsA-HA1/*L. casei* is capable of inducing robust protective immune responses that are potent enough to block virus replication in vivo.

## Discussion

Mucosal immunity plays a significant role in defense against influenza virus infections, and the induction of effective mucosal immune response is the primary objective of vaccination. However, mucosal barriers typically respond to exogenous antigens with tolerance instead of immune activation, making it difficult to elicit local immune responses through immunization. Consequently, significant efforts have focused on developing potential adjuvants and vaccine delivery vectors for mucosal application in order to overcome these challenges [47].

Recombinant lactic acid bacteria (LAB) are increasingly being used as a potential carrier for mucosal vaccine delivery due to their ability to adhere to mucosa surface [5, 36, 48], intrinsic immunomodulatory properties [49-52], and feasibility in displaying heterologous antigens on the surface [33, 34, 36]. The display of heterologous antigens on the bacterial surface is usually achieved by genetic fusion with a bacterial transmembrane anchoring protein [53]. In this study, we explored the potency of recombinant *L. casei* as a surface-expression system and delivery vector for a mucosal vaccine against AIVs. Specifically, we replaced the domain (residues 26 to 42) that protrudes from the core helix structure of the *Bacillus subtilis* pgsA protein with H5N2-derived HA1 antigen through a translational fusion. Subcellular fractionation and flow cytometry analyses confirmed the successful expression and display of the HA1 antigen at the *L. casei* cell surface (Fig. 1B and 1C), making it a promising mucosal vaccine candidate

HA is a classical type I membrane glycoprotein that plays key roles in viral adsorption and membrane fusion. This protein stimulates the production of functional antibodies and is the primary antigen in many preclinical studies [54]. During virus replication, the HA protein is initially translated as a single polypeptide precursor (HA0), which is later cleaved by host trypsin-like proteases into two subunits, HA1 and HA2 [55]. The HA1 subunit forms a membrane-distal globular head that contains the receptor-binding domain (RBD), and most of the antigenic regions are recognized by neutralizing antibodies [56, 57]. In this study, we proved that oral or intranasal administration of recombinant pgsA-HA1/*L. casei* in mice induced significant HA1-specific neutralizing antibodies against the H5N2 strain (Fig. 2E and 2I), elicited potential cellular immune responses in both local and systemic compartments (Fig. 3) and provided sufficient protection against the mouse-adapted H5N2 virus challenge (Fig. 4). Also, our results are in line with the previous studies in which *L. lactis* and *L. plantarum* surface-displayed HA1 antigens conferred the potential protection of mice against the lethal H5N1 and H1N1 virus infections, respectively [58, 59].

The route of vaccine administration is an important parameter that, can significantly affect the quality and quantity of the immune responses [60]. In this study, mucosal administration with recombinant *L. casei* in mice elicited not only systemic IgG but also local IgA, in mice, which is in agreement with previous observations [36, 61]. Our results indicated that intranasal administration of the vaccine enhanced HA1-specific IgA antibody responses in the respiratory tract, while oral inoculation triggered a more potent IgA antibody response in the intestinal surface. The magnitude of HA1-specific IgA antibodies varied between oral and intranasal immunization, possibly due to the distinct way in which antigens are processed and presented to the immune cells in two mucosal compartments. However, the respiratory tract is the primary site of influenza infection, necessitating a high IgA concentration in the lungs or nasal surfaces. This is why the mice subjected to intranasal immunization fared better after influenza infection (Fig. 4).

Antigen-induced cell-mediated immune responses are essential for host protection against AIV infection. Lactobacilli have been shown to promote the secretion of pro-inflammatory cytokines such as IL‐6, IL‐12, and TNF‐α, which further stimulate NK cells to secrete IFN‐γ to enhance cytotoxic CD8 T lymphocyte (CTL) responses [62]. Our data suggest that intranasal inoculation of pgsA-HA1/*L. casei* stimulated a tremendous increase in antigen-specific systemic IFN-γ levels compared to oral administration of the same antigen. Additionally, we observed that oral and intranasal administration of *L. casei* surface-exposed HA1 induced different levels of local IL-4 cytokines in secondary lymphoid organs, likely due to differences in mucosa-associated lymphoid tissue (MALTs), where the immune responses to the antigen are initiated [63]. These results suggest that our L. casie-based mucosal vaccine potentiates CD4^+^ Th1/Th2-skewed, possibly in combination with CD8^+^ T-cells to the HA1 antigen. Further investigation is needed to understand the proliferation of effector and memory CD4^+^/CD8^+^ T cell subsets activated by the pgsA-HA1/*L. casei* vaccine.

*Lactobacilli* are low-toxic commensal organisms, have well-established safety records, and are held Generally Recognized as Safe (GRAS) and Qualified Presumption of Safety (QPS) status. This allows for the design of vaccines that have minimal side effects while preserving their inherent adjuvant properties to stimulate immune responses [64]. Mucosal administration of LAB vaccines is capable of stimulating both systemic and mucosal immune responses [65]. This non-invasive method is a preferable alternative for individuals who are needle-averse. LAB elicit minimal immune responses against themselves while inducing high levels of immune responses to foreign antigens following uptake via the mucosal immune system [66]. They are also efficiently transported to Peyer's patches, which are the inductive sites of the mucosal immune system, meeting the requirements of a delivery system for mucosal immunization [67]. However, there are several safety concerns associated with the practical application of recombinant LAB vaccines, including the potential release of genetically modified organisms into the environment and the development of antibiotic resistance [68].

In summary, our findings revealed that administering recombinant pgsA-HA1/*L. casei* via mucosal routes induced local and systemic protective immune responses that protected mice against infection with the H5N2 virus. These results suggest that, recombinant pgsA-HA1/*L. casei* potential as a mucosal vaccine candidate against future outbreaks of H5N2 virus. However, to ensure the broad applicability of this vaccine studies are necessary to determine whether it can provide similar protective efficacy against other H5Nx viruses.
